# Evaluating the impact of heat stress as measured by temperature-humidity index (THI) on test-day milk yield of small holder dairy cattle in a sub-Sahara African climate

**DOI:** 10.1016/j.livsci.2020.104314

**Published:** 2020-12

**Authors:** C.C. Ekine-Dzivenu, R. Mrode, E. Oyieng, D. Komwihangilo, E. Lyatuu, G. Msuta, J.M.K. Ojango, A.M. Okeyo

**Affiliations:** a Animal Biosciences, International Livestock Research Institute, Nairobi, Kenya; b Animal and Veterinary Science, Scotland Rural College, Edinburgh, United Kingdom; c Tanzania Livestock Research Institute, Tanzania

**Keywords:** Heat stress, Temperature humidity index, Milk loss

## Abstract

This study evaluates the effect of heat stress on milk production and describes the pattern of response of milk yield to increasing heat load, using temperature-humidity index (THI) on test-day milk records of small holder dairy cattle herds in the sub-Saharan African climate of Tanzania. Climate data obtained from aWhere, an agricultural weather data platform (http://www.awhere.com) was analysed with 14,367 first lactation test day milk records of 3511 dairy cows collected between 2016 and 2019. THI was calculated from daily maximal temperatures and daily minimum humidity. Three sets of analysis were performed. In the first and second analysis, two mixed effect repeatability models were fitted with THI treated as a categorical variable grouped into 5 classes (THI1= [61 - 66], THI2= [67 - 71], THI3= [72 - 78], THI4=[79 - 81], THI5=[82 - 86]), to obtain least squares estimates of THI effect on milk production, and as a continuous variable within THI classes to identify THI thresholds at which milk yield started to decline. In the third analyses, one quadratic polynomial regression (POL) and three regression spline functions namely piecewise linear spline function (PLF), natural splines function (NSF) and cubic splines function (CSF) were fitted to determine the average effect of THI on milk yield in the population and describe the pattern of response of milk yield to increasing head load.

The results show that heat stress reduced milk yield by 4.16% to 14.42% across THI groups, with daily milk yield being the highest in THI1 (7.40±0.39 litres) and the lowest in THI4 (6.33±0.32).

Regression coefficients within groups showed significant daily milk yield decrease in THI2 (-0.09) and THI3 (-0.06), but not for other THI classes, indicating that cows experienced heat stress between THI values of 67 and 76 and milk loss plateaued afterwards, suggesting that the animals acclimatized to heat stress conditions beyond THI value of 76. At the population level, THI and its squared term were significantly negatively and positively (-0.61, 0.004) associated with milk production, indicating a non-linear relationship between milk yield and THI. The CSF model showed better goodness of fit and predictive ability than other models for predicting future population response of milk yield to heat stress in small holder dairy farms in Tanzania.

Herd management strategies and animal husbandry measures are needed in small holder dairy farms in Tanzania to minimize the impact of heat stress on milk yield and income of the farmers.

## Highlights

• This study evaluates the effect of heat stress on milk production and describes the pattern of response of milk yield to increasing heat load, in small holder dairy farms in sub-Saharan Africa.
• Milk yield showed a W-shaped pattern of response across the THI scale.
• Cows experienced heat stress in the THI window between THI values of 67 and 76.
• Milk loss plateaued beyond THI value of 76 suggesting that the animals acclimatized to the heat stress conditions despite the initial heat load shock.

## 1 Introduction

Lactating dairy cows dissipate a large amount of heat from metabolic processes associated with maintenance and milk production (West et al., 2003). Exposing lactating dairy cows to high ambient temperature and high relative humidity for extended periods decreases their ability to dissipate heat generated from both metabolic processes and heat gained from the environment, making them susceptible to heat stress (Gantner et al., 2015; Pragna et al., 2017). To decrease its heat load, the cow reduces its feed intake and consequently milk production (Silanikove, 2000; Summer et al., 2019). The severity of heat stress effects on cows will increase as climate change and global warming escalates and this impact will be felt more among producers with a subsistence economy in tropical regions of developing countries (Hernández et al., 2011; Herrero et al., 2010).

Even though dairy cattle in parts of sub-Saharan Africa are routinely subjected to high ambient temperature and relative humidity, quantification of the effect of heat stress on milk production under sub-Saharan climatic conditions is very limited.

The objectives of this study were to evaluate the effect of heat stress on milk production and describe the pattern of response of milk yield to increasing heat load as measured by temperature-humidity index (THI) on test day milk yield in small holder dairy cattle populations in the sub-Saharan African climate of Tanzania.

## 2 Materials and methods

### 2.1 Data

This study was carried out as part of a wider project called African Dairy Genetic Gains^1^ which is developing and testing a multi-country genetic gains platform using smallholder dairy cattle farm performance and genomic information. The project is being implemented in partnership with several national and international institutions and is led by the International Livestock Research Institute (ILRI). 14,367 first lactation test day milk records of 3511 dairy cows, collected monthly, between January 2016 to November 2019, using the open data kit software (ODK) from 2927 small holder dairy farms in Tanzania were used in this study. The cows were housed in sheds made of different construction materials including cement, wood and corrugated iron sheets. At each recording, the cows had morning and evening milk yields measured. Only cows with at least 2 test day records within 400 days in milk were included in the analysis.

Climate data was obtained from aWhere (http://www.awhere.com), an agricultural weather data platform using GPS coordinates of the farms at 10 km grid cell resolution. This included daily maximum and minimum temperature (°C) and relative humidity (%). Daily THI was calculated by applying the National Research Council (1971) formula as follows:

THI=(1.8×Tmax+32)−[(0.55−0.0055×RHmin)×(1.8×Tmax−26.8)]

where Tmax is the maximum daily temperature and RHmin is the minimum daily relative humidity. Among other THI models developed to study the impact of heat stress on performance of animals, this model was considered the most appropriate for the equatorial climate of Tanzania. Ravagnolo et al. (2000) reported that combining daily temperature and humidity values in this manner to define THI index showed a slightly superior goodness of fit than other combinations under the hot and humid conditions of Georgia, United States.

Each THI was calculated using a 4-day average of temperature and relative humidity obtained from measures on the test day and 3 days prior to the test day. This range enables the determination of the prolonged effect of heat stress on milk production on a given day (Bohmanova et al., 2008; Hammami et al., 2013)

### 2.2 Statistical models

Three sets of analyses were carried out using the R statistical package (R Core Team, 2019). In the first analysis aimed at estimating the effect of THI on daily milk production and depicting the shape of milk yield response to heat stress in the population, THI was grouped into 5 classes, THI1= [61 - 66], THI2= [67- 71], THI3= [72 - 78], THI4=[79 - 81], THI5= [82 – 86] and treated as a categorical variable in a mixed linear regression repeatability model fitted to obtain least squares estimates of THI effect on milk yield. Pairwise comparison of least square means between THI classes were computed to test for significant differences in milk yield between THI classes.

The aim of the second set of analysis was to quantify the rate of decline of milk production across THI classes i.e. quantify change in milk yield in response to increasing heat load. This was achieved by splitting the data into 5 sets according to THI classes and calculating linear regression coefficients within each THI class interval in a mixed linear regression repeatability model in which THI was treated as a continuous variable.

In the third set of analyses, aimed at determining the average effect of THI on milk production and describe the pattern of response of milk yield to increasing headload, four models, one quadratic polynomial regression (POL) and three regression spline functions namely: piecewise linear spline function (PLF), natural splines function (NSF) and cubic splines function (CSF) were used. The regression spline models were fitted using linear and cubic polynomial functions in each of the 5 THI class bins with 4 knots at THI 66,71,78, and 81 respectively, based on THI class intervals. The splines package in the R statistical software was used for this analysis. Models were compared for goodness of fit using the Akaike information Criterion (AIC), and predictive ability for future data using root mean square error (RMSE) and R squared (Rsq). RMSE measures model prediction error, which is the average of differences between observed and predicted values while Rsq measures squared correlation between observed and predicted values. The data was randomly split into a training set (80%) for building the prediction model and test set (20%) for evaluating the model.

All models included age at calving, days in milk, region, breed as declared by the farmer and herd year effects. The mixed linear regression repeatability models fitted had a general form as follows:

(1)) $$y_{\text{ijklmno}}=\text{ag}e_{i}+\text{di}m_{j}+\text{bree}d_{k}+\text{TH}I_{l}+hy_{m}+{\text{c}}_{\text{n}}+e_{\text{ijklmno}}$$

where y_ijklmno_ is the o^th^ test-day record (milk yield in liters) of the n^th^ cow; age_i_ is the i^th^ age at calving; dim_j_ is the j^th^ days in milk (dim) class; breed_K_ is the k^th^ breed class; THI_l_ is the l^th^ THI class, hy_m_ is the effect of the m^th^ combination of herd and year of recording, c_n_ is the nth cow effect and e_ijklmno_ is the residual error.

The quadratic regression and spline models had a general form as follows:

(2)) $$y_{\text{ijklmno}}=\text{ag}e_{i}+\text{di}m_{j}+\text{bree}d_{k}+f\left(T\right)+hy_{m}+c_{n}+e_{\text{ijklmno}}$$

where f(T) is a function of THI which differed between the models. For the regression spline models,

$$f\left(T\right)=\;\sum_{j=1}^{q}bj\left(K-THI\right)$$

where q is the order of splines defined over the number of knots. The covariable (K-THI) was set to zero for THI values below the specific knot threshold (K) and bj represents the jth spline coefficient.

For the quadratic regression model,

$$f\left(T\right)=\;\sum_{j=1}^{q}bj\left(THI\right)$$

where b_j_ is the polynomial regression coefficients for the linear (*j* = 1) and quadratic (*j* = 2) effects of THI and q is the polynomial degree.

## 3 Results and discussion

### 3.1 Description of the data

**Fig. 1:**
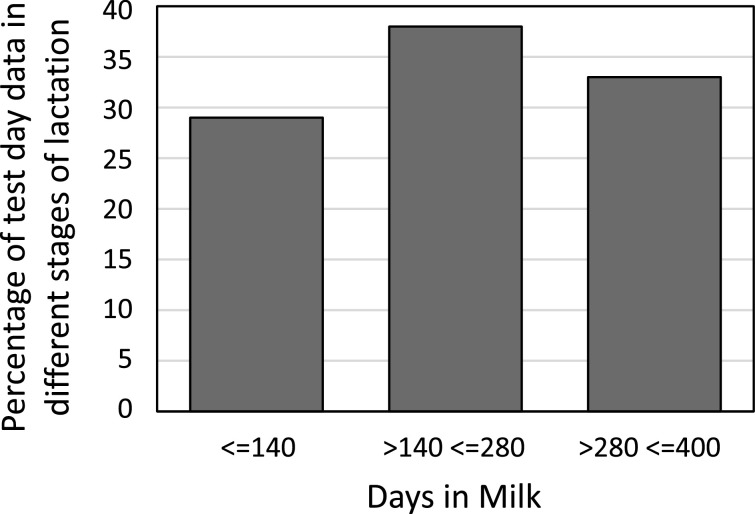
Distribution of milk records across the different stages of lactation.

**Fig. 2:**
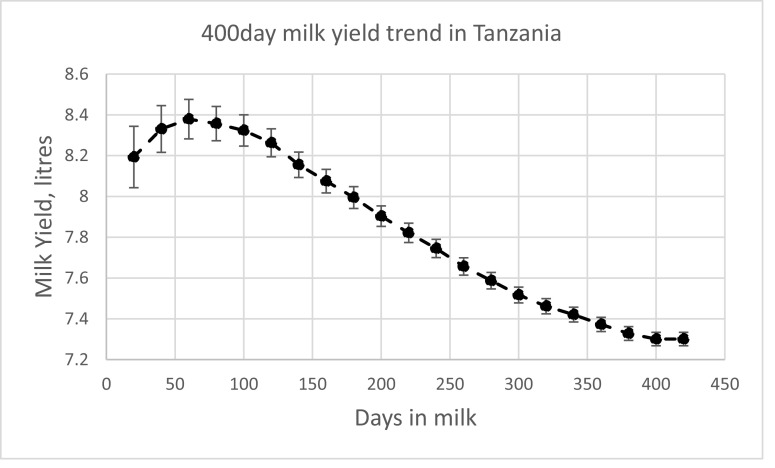
Milk yield trend for 400-day milk production.

A summary of the mean, standard deviation and range of test-day milk yield including a summary of climate data characterizing environmental conditions during the period of this study within THI groups is presented in Table 1 and shows the wide range of measured milk yields, temperature, relative humidity and THI values. Milk records used for this analysis was well distributed across the different stages of lactation (Fig. 1). Daily milk yield followed the pattern of a normal lactation curve and peaked at 60 days (Fig. 2). There was between 1 and 8 first lactation cows per farmer and between 2 and 15 records per cow and 2 to 36 records per farm.

**Table 1:** Mean, standard deviation (SD) and range of climate data and test-day milk yield during the period of this study.

		THI group				
		THI1 [61,66]	THI2 [67,71]	THI3 [72,76]	THI4 [77,81]	THI5 [82,86]
Temp, ^O^C						
	mean	20.37	23.83	26.78	29.91	32.66
	SD	5.18	5.38	5.23	5.20	4.48
	range	7.35 - 22.23	8.3 - 27.83	11.25 - 31.13	15.63 - 34.58	21.3 - 35.2
RH%						
	mean	87.74	91.16	93.54	93.36	92.80
	SD	24.96	25.30	24.57	22.59	18.37
	range	22.98 - 100	13.3 - 100	16.18 - 100	26.13 - 100	44.33 - 99.25
THI						
	mean	65.41	69.85	73.93	78.41	83.20
	SD	0.85	1.20	1.40	1.33	1.09
	range	61.13 - 66.46	66.58 - 71.50	71.50 - 76.50	76.50- 81.48	81.50 - 86.36
Milk, liter						
	mean	7.67	7.64	7.43	6.52	5.99
	SD	4.07	4.03	3.84	3.41	3.03
	range	1.5 - 18	0.5 - 19	0.25 - 19	0.5 - 19	0.5 - 18

**Fig. 3:**
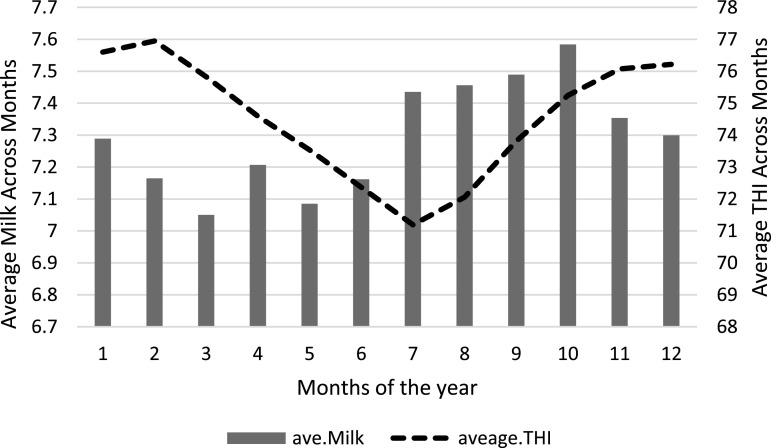
Trend in milk yield and Temperature Humidity Index (THI) across months.

Variations in milk yield and THI across all months of the year are shown in Fig. 3. Milk yield decreased between November to March and then increased between April to October. The opposite trend was observed in the THI values i.e. as THI increased, milk yield decreased and as THI decreased, milk yield increased. Sharp decline in milk yield was observed when THI reached 77 and improvement was seen as THI dropped to 71. These seasonal differences in THI values and the associated changes in milk yields could be directly associated with changes in the environmental climate over the months, and such environmental changes also results in fluctuations in quantity and quality of feed offered to the cows.

At the same temperature, the heat load on the animal as indicated by the THI is higher at higher humidity levels, than at lower humidity levels (Table 2). This is because cattle dissipate excessive heat by sweating and when it is humid, their natural ability to dissipate heat by sweating and panting is lowered, leading to heat stress (Habeeb et al., 2018; Marai and Habeeb, 1998).

**Table 2:** THI calculated from maximum air temperature and minimum relative humidity showing that various combinations of temperatures and humidity values yield the same THI.

	Relative Humidity,%																			
Temperature °C	13	17	19	21	24	28	30	36	39	40	44	46	53	59	61	68	72	74	77	80
17																				
19								64	64	64				65						
20								65	65	65		65								
21						65		66	66	67	67	67		68	67	69	69			
22							67	67	68	68	68	68	69		68		70			
23			67		68		68	69	69	69	69	70	70	71	71	72	71			
24		68	68	68	69	69	69	70	70	70	71	71	72	72	72	73	73			
25	69	69		69	70	70	70	71	71	72	72	72	73	74	74	74		74		
26		69	70	71	71	71	72	72	73	73	73	73	74	75	75	76				
27		71	71	71	72	72	73	73	74	74	74	75	76	76	76	77			77	
28		72	72	72	73	73	74	75	75	75	76	76	77	78	78	78	79			
29		72	73	73	74	74	75	76	76	76	77	77	78	79	79		80	80		82
30				74	74	76	76	77	77	78	78	78	80	80	81		82			
31						77	77	78	79	79	79	80	81	82	82	83				
32						77		79	79	80	80	80	82	83	84					
33							79	80		80	81	81	83	85	85					
34							80				83		85	85	86					
35													86							

Blank cells indicate that the combination of temperature and humidity was not observed in our data.

### 3.2 Response of milk yield to THI

**Fig. 4:**
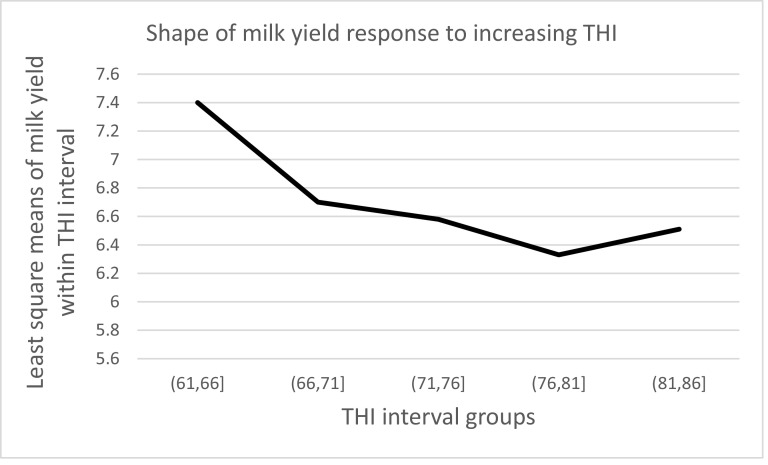
Shape of milk yield response to increasing heat load under model1. Least square means obtained from model 1 with THI treated as a categorical variable.

Least square means, standard errors, and pairwise comparison of milk yield within THI groups for the effect of THI on milk production is presented in Table 3. The results show that heat stress significantly affected daily milk production. Daily milk yield in THI1 [61,66] was significantly higher (7.40±0.39 litres) at *P*< 0.05 than in other groups. Milk yield in THI 4 [79–81] was the lowest (6.33±0.32). Heat stress reduced milk yield by 4.16% to 14.42% across THI groups. Milk yield showed a W-shaped pattern of response across the THI scale (Fig. 4).

**Table 3:** Least square means (±Standard Error) for milk yield within THI groups and pairwise comparison for milk yield across groups from model 1 in the first set of analysis.

	Least square means (±SE) for milk yield within THI groups and pairwise contrasts of differences (P-value) between THI groups				
	THI1	THI2	THI3	THI4	THI5
	(61,66]	(66,71]	(71,76]	(76,81]	(81,86]
	7.40±0.39	6.70±0.32	6.58±0.32	6.33±0.32	6.51±0.34
THI1 [61,66]		0.70 (0.012)	0.83(0.002)	1.07 (<0.0001)	0.89(0.003)
THI2 [66,71]			0.13(0.098)	0.37(<0.0001)	0.19(0.545)
THI3 [71,76]				0.24(<0.001)	0.06(0.984)
THI4 [76,81]					−0.18(0.383)

Regression coefficients calculated within THI groups are presented within Table 4. Results showed that change in milk yield per unit increase in THI was negative and significant for THI2 (−0.09) and THI3 (−0.06), which means that for every unit increase in THI, milk yield decreased by 0.09 and 0.06 litres respectively within these 2 groups. Regression coefficients for THI groups 1, 4 and 5 were 0.09, 0.05 and −0.01 respectively, and were not statistically significant at *P*<0.05. This result suggests that cows in this population entered heat stress after THI value of 66. Milk loss seemed to plateau beyond THI value of 76 [THI3] since the regression coefficients for milk yield was not significant beyond this point. The plateauing of milk yield beyond THI value of 76 might be because the animals acclimatized to the heat stress conditions beyond this THI value. Indeed, Horowitz (2001) explains that when exposed to prolonged heat load, animals can acclimatize to the heat stress through the process of acclimatory homeostasis which is characterized by a decrease in growth hormone, catecholamine and glucocorticoid levels. The altered endocrine status acts to lower circulating levels of thyroxine (T4) and triiodothyronine (T3) resulting in reduction in basal metabolic rate and heat production (Johnson, 1980; Yousef, 1987). For lactating cows, such changes in hormonal profiles results in reduction in milk yield. There is therefore a lag period before acclimatory homeostasis is reached. During the lag period the animals is stressed and milk production is reduced.

**Table 4:** Change in milk yield (α), within THI intervals due to heat stress estimated using model 1 in the second set of analysis with data split into 5 categories according to THI class.

	THI1 (61,66]	THI2 (67,71]	THI3 (72,76]	THI4 (77,81]	THI5 (82,86]
α	0.09	−0.09	−0.06	0.05	−0.01
Standard.Error	0.25	0.04	0.02	0.04	0.08
P-value	0.72	<0.01	<0.01	0.20	0.88

At a similar THI threshold as in this study, Gantner et al. (2015), in Eastern Croatian Holstein cattle, observed a significant drop in daily milk yield when THI exceeded 67 and milk yield decreased from 0.24 to 0.72 kg/day. In the Mediterranean climate of Tunisia, Bouraoui et al. (2002) also observed a 21% decrease in milk production when THI value increased from 68 to 78 for lactating Holstein Friesian cattle. Moreover, Zimbelman et al. (2010), Bernabucci et al. (2010) and Collier et al. (2012) reported significant losses in milk yield at THI 68 in Holsteins cows in Arizona and Italy. In Southern Africa and Namibia however, Du Preez et al. (1990) reported that the performance of dairy cows was affected, and milk production inhibited by heat stress when THI values were higher than 72. In the USA, Bohmanova et al. (2007) reported different THI threshold values for different regions (72 in Athens, Georgia, and 74 in Phoenix, Arizona). In Holstein cows reared in the Mamara region of Turkey, Duru et al. (2018), found that adverse effect of heat stress on milk yield could be noticed at THI 65 but milk yield declined irreversibly after THI 70. Brugemann et al. (2012) assessed the impact of heat stress on test-day records of Holstein cows in the state of Lower Saxony, in three different regions characterized by different production systems using different heat stress indicators. THI Thresholds for THI index calculated from mean and maximum temperatures as was done in this study were 67 in the maritime region; 73 for the pasture-based system, and 74 for the region characterised by crop production. Zare-Tamami et al. (2018) reported that milk yield reduced significantly in Northern Iranian Holstein cows for THI values that were higher than 60.

These different THI thresholds can be achieved with a range of different temperature and humidity combinations as shown in Table 2 and therefore causes of decline in milk production differs between climates and environments. In hot and humid climates, evaporative cooling, which is an effective mechanism for alleviating heat stress is compromised, and milk production is adversely affected at lower temperatures than cows can otherwise comfortably accommodate. On the other hand, cows in a semiarid climate, can cope with higher absolute temperatures before they show similar decreases in productivity as observed in cows kept in hot and humid climates where temperatures are lower, but humidity is much higher (Bohmanova et al., 2007).

The effect of heat load on average milk yield in this population (Table 5) shows that daily milk yield per cow per day was significantly negatively (−0.61) associated with the linear THI term and significantly positively (0.004) associated with its squared term. This means that there is no unique slope (first derivative of the quadratic function ⌈slope=−0.61+(2*0.004)*THI⌉) that applies throughout the THI interval observed in this study since there is a nonlinear relationship between THI and milk yield. Change in milk yield related to a unit increase in THI depends on where one starts and is continuously changing throughout the THI interval. A unit increase in THI will reduce the slope of milk loss by 0.008 (2nd derivative of the function) such that the effect of THI on milk yield is diminished for larger THI values. The response curve has a minimum (given that the 2nd derivative is positive) occurring at 76.25 units of THI which is the turning point of the curve, as it changes directions from decreasing to increasing.

**Table 5:** Average effect of THI at the population level estimated using the POL function under model 2 in the third set of analysis.

	THI	THI^2
Regression Estimate	−0.61	0.004
Standard.Error	0.173	0.001
P-value	< 0.01	< 0.01

No large differences for goodness of fit and predictive ability was observed between models describing the pattern of response in milk yield to increasing heat loads (Table 6) in this population. Nonetheless, the CSF model provided slightly better statistical features in terms of lower AIC, RMSE and higher Rsq compared to other models and would be preferred in predicting future population response of milk yield to heat stress in small holder dairy farms in Tanzania.

**Table 6:** Model comparison on goodness of fit to data (AIC) and predictability of the model (RMSE and Rsq) using model 2 in the third set of analysis.

Model	AIC	RMSE	Rsq
POL	66,599.71	2.354731	0.619549
LPF	66,604.89	2.354029	0.619788
NSF	66,578.08	2.353699	0.61988
CSF	66,570.65	2.353056	0.620083

AIC – Akaike information criterion, RMSE – Root mean squared error (measures model prediction error, which is the average of differences between observed and predicted values), Rsq – R-Squared (measures the squared correlation between the observed and predicted values), POL – Quadratic polynomial Function, PLF - Piecewise linear spline function, NSF - Natural splines function, CSF - Cubic splines function (CSF).

The average estimate of milk decline obtained in the present study varies from those reported by others. Wildridge et al. (2018), reported an average of 0.15 kg decline in milk yield per unit rise in THI using data obtained from 6 pasture-based farms in Australia. Brugemann et al. (2012), observed a decline of 0.08 kg, 0.17 kg, and 0.26 kg respectively per unit increase in THI in three different dairy production regions in the state of Lower Saxony, Germany. In Vojvodina (Serbia), Konyves et al. (2017) evaluated the relationship between THI and milk yield in Holstein Friesian cows across different seasons of the year, and found that milk yield decreased by 0.011 kg, 0.108 kg and 0.046 kg, respectively in spring, summer and autumn for every unit rise in THI. In Italian Holstein cows, Bernabucci et al. (2010) observed a decrease of 0.27 kg milk per day for each THI unit increase above 68 while Bouraoui et al. (2002) reported that milk yield declined by 0.41 kg, per cow, per day, per unit rise in THI values above 69 in Tunisian Holstein-Friesian cows.

Variations in THI effects reveal differences in environmental and climatic conditions across studies, but also differences in breed, farm management practices and statistical methods used to analyze the data. We have used polynomial functions, rather than the broken line function commonly used to describe productive response to increasing heat loads in animals (Carabano et al. 2014), to capture the pattern of response to increasing heat load in animals from small holder farm settings in this study as it was a better fit to the data. The traditionally used broken line function assumes that milk yield remains constant within a thermoneutral zone, where no response to increasing temperatures is observed and that after a certain threshold (the breaking point) which marks the start of heat stress, milk yield decreases linearly (Misztal, 1999; Kadzere et al., 2002). This assumption may however be too simplistic and not offer the flexibility needed to model data in such a way as to account for the more complex production systems that characterize small holder dairy farming systems in tropical regions of developing countries.

The reduction in milk production during heat stress may be attributed to decreased nutrient intake and uptake by the portal drained viscera of the cow (Gantner et al., 2011; Beede and Collier, 1986; Mohammed and Johnson, 1985). Schneider et al. (1988) observed that heat stressed dairy cows in a chamber experiment consumed less feed (13.6 vs. 19.4 kg/day), more water (86.0 vs. 81.9 l/day) and produced less milk (16.5 vs. 20.0 kg/day) than cows in a thermal neutral environment. Johnson et al. (1963) reported that milk yield and dry matter intake (DMI) exhibited significant decline (by 1.8 and 1.4 kg) when maximum THI reached 77. Such reductions in milk yield could be exacerbated by reduced feed quality, water availability and intake. Under smallholder systems, water is often limiting and more so during the hot and dry months of the year. If no additional water is provided to compensate for the additional sweating and heat increments that are associated with digesting poorer quality feed, then higher milk yield decreases are expected. When poorer and little feed and water is on offer, production would naturally decrease, as cows begin to draw on their body reserves to meet maintenance needs, besides that for milk production.

With continued genetic improvements for increased milk yield, there is a chance that dairy cattle will become even more susceptible to heat stress in the face of increased climate change threats, unless heat tolerance is included in the selection index.

## 4 Conclusion and recommendations

In conclusion, results from this study showed that heat stress measured by THI significantly reduced daily milk production in this population with milk yield showing a W-shaped pattern of response with increasing heat load. The cows in this population entered heat stress after THI value of 66 with milk loss plateauing beyond THI value of 76. Acclimatization of animals in this system to the prevailing heat stress conditions might explain the plateauing of milk loss. The cubic spline function provided a slightly superior goodness of fit and predictive ability for describing changes in milk yield along the range of studied temperature and relative humidity in this population.

To mitigate against the imminent impact of heat stress in small holder dairy farming systems, both short term and longer-term precautionary measures are required. In the short term, the goal should be to encourage small holder farmers to put in herd management and animal husbandry measures that effectively protect the animals from too much heat. Such heat mitigation measures would include use of shades in form of trees, hay or straw shades or roofing with corrugated sheets painted with reflective coating on top (e.g. white paint) to reflects sunlight and use of higher ceilings in buildings to reduce temperature absorbed inside, provision of adequate feed and water at all times and increasing the use of concentrate and less use of roughage within limits of maintaining proper rumen function in hot weather.

In the longer term, identifying sires and dams that carry genes which confer better tolerance to heat stress and using them for breeding future dairy animals for smallholder farming systems in sub-Saharan Africa could be a more sustainable strategy.

Additional studies to explore the related wider effects of heat stress on milk quality, fertility, reproduction and growth in small holder dairy cattle in sub-Saharan Africa are needed.

## Author statement

C.C. Ekine-Dzivenu Writing Formal analysis, Writing- Original draft preparation.

R. Mrode writing- Reviewing and Editing, Supervision

E.Oyieng Data Curation

D. Komwihangilo Project administration

E. Lyatuu Project administration

G. Msuta Project administration

J. M.K. Ojango writing- Reviewing and Editing,

A.M. Okeyo, writing- Reviewing and Editing, Funding acquisition

## Declaration of Competing Interest

None.

## Appendix Supplementary materials

Image, application 1
